# 

**Published:** 1897-01

**Authors:** 


### Central Texas Medical Association.

Programme of the seventh semi-annual session of the Central Texas Medical Association, to be held at Waco, Texas, Tuesday and Wednesday, January 12-13, 1897.

DEAR DOCTOR:—If you are a member of the "Central Texas Medical Association" your interest demands your presence and participation in the seventh semi-annual session, at Waco, Texas, January 12, 13, 1897. If you are not a member and wish to associate yourself with your professional confreres in contributing to the general fund of professional knowledge, you are cordially welcomed to membership in this Society. The programme presents a splendid medical and surgical menu, and your contribution to professional thought and advancement will improve the feast. Doctor, come and help us make the first meeting of 1897 a prophet of greater success and usefulness in our chosen profession.

Cordially and fraternally your,

MARVIN L. GRAVES, M. D., Secretary.

J. CROSBY SHAW, M. D., President.

PAPERS.

1. Pneumonia: A. J. Weathered, Waco. Discussion by J. D. Foster, of Reisel, and T. N. Clark, of Reagan.

2. Metritis: W. E. Brown, of Gatesville. Discussion by J. D. Moore, of Eddy, and R. F. Minnock, of Waco.

3. Prostatitis: W. A. Howard, of Waco. Discussion by M. D. Knox, of Hillsboro, and W. M. Shankle, of Chilton.

4. Retained Placenta after Abortion and Its Treatment: W. C. Blalock, of Kosse. Discussion by Daniel Parker, of Calvert, and R. P. Talley, of Waco.

5. Anæsthetics, and the Best Modes of Administration: Taylor Hudson, of Belton. Discussion by W. R. Blaylock, of McGregor, and O. I. Halbert, of Waco.

6. Tubercular Meningitis: J. D. Law, of Salado. Discussion by W. W. Greer, of Cameron, and S. M. Jenkins, of Summer's Mill.

7. Otorrhœa in Children: Joseph R. Anderson, of Waco. Discussion by R. J. Pope, of Jonesboro, and C. Guy Reilly, of Waco.

8. Primary Syphilis, Its Diagnosis and Treatment: R. W. Noble, of Temple. Discussion by W. B. Newland, of Gatesville, and W. T. Baird, of Dallas.

9. Prevention and Treatment of Opthalmia Neonatorum: E. C. Gordon, of Lott. Discussion by W. F. Cole, of Waco, and R. C. Nettles, of Marlin.

10. Eczema Seborrhœa: J. B. Shelmire, of Dallas. Discussion by U. H. Nixon, of Killeen, and N. A. Olive, of Waco.

11. Disease of Accessory Sinuses; Diagnosis and Treatment: J. M. Woodson, of Temple. Discussion by A. C. Scott, of Temple, and H. L. Taylor, of Waco.

12. Acute and Chronic Cystitis; Diagnosis and Treatment: H. C. Ghent, of Belton. Discussion by J. C. J. King, of Waco, and T. J. Hubbert, of Hico.

Voluntary Papers.

Individual Reports of Cases.

Miscellaneous Business.

"X Ray Lecture." Prof R. S. Hyer, Professor of Natural

Science in Southwestern University, will lecture on the X Rays, before the Association, aud will photograph a number of patients with bullets, foreign bodies and broken bones in the body. The photographs will be shown in a few minutes after they are taken.

